# Supplementary material for: NHANES 2011–2014 Reveals Cognition of US Older Adults may Benefit from Better Adaptation to the Mediterranean Diet
Source: Nutrients. 2020 Jun 29;12(7):1929. doi: 10.3390/nu12071929 (PMC7399952; doi:10.3390/nu12071929)
Supplement: Supplementary file 1 [file nutrients-12-01929-s001.pdf]

```
### ----------NHANES 2011-2012 data correlates with the letter "G"---------- ###
library(nhanesA)

##load NHANES cog data
cog1112 <- nhanes('CFQ_G')

##load NHANES 2011-2012 nutrient data
nutrients1_1112 <- nhanes('DR1TOT_G')
nutrients2_1112 <- nhanes('DR2TOT_G')

foods1_1112 <- read_sas(file.choose())
foods2_1112 <- read_sas(file.choose())

coded.food1.1112 <- nhanes('DR1IFF_G')
coded.food2.1112 <- nhanes('DR2IFF_G')
foodcode1112 <- nhanes('DRXFCD_G')

#select only olive oil from individual food data
coded.food1.1112 <- coded.food1.1112[c(1, 18, 20)]
coded.food1.1112 <- coded.food1.1112[ which(coded.food1.1112$DR1IFDCD ==
82104000), ]

coded.food2.1112 <- coded.food2.1112[c(1, 18, 20)]
colnames(coded.food2.1112)[colnames(coded.food2.1112)=="DR2IFDCD"] <-
"DR1IFDCD"
colnames(coded.food2.1112)[colnames(coded.food2.1112)=="DR2IGRMS"] <-
"DR1IGRMS"
coded.food2.1112 <- coded.food2.1112[ which(coded.food2.1112$DR1IFDCD ==
82104000), ]

#combine coded food DFs
coded.food1112 <- rbind(coded.food1.1112, coded.food2.1112)
colnames(coded.food1112)[colnames(coded.food1112)=="DR1IGRMS"] <- "OliveOil.g"
coded.food1112 <- coded.food1112[-c(2)]
coded.food1112 <- stats::aggregate(. ~ SEQN, data = coded.food1112, FUN = "mean")

##2011-2012 nutrient data processing
names(nutrients1_1112) <- gsub("DR[1-9]", "", names(nutrients1_1112))
nutrients1_1112 <- nutrients1_1112[, names(nutrients1_1112) %in% nutr_keepers]
names(nutrients2_1112) <- gsub("DR[1-9]", "", names(nutrients2_1112))
nutrients2_1112 <- nutrients2_1112[, names(nutrients2_1112) %in% nutr_keepers]
nutrients1112 <- rbind(nutrients1_1112, nutrients2_1112)
nutrients1112 <- data.frame(apply(nutrients1112, 2, as.numeric))
nutrients1112 <- nutrients1112[!is.na(nutrients1112$TSFAT), ]
nutrients1112 <- stats::aggregate(. ~ SEQN, data = nutrients1112, FUN = "mean")
```

```
##load NHANES demographic data
demo_1112 <- nhanes("DEMO_G")

##2011-2012 change food var names
names(foods1_1112) <- gsub("DR[1-9]", "", names(foods1_1112))
names(foods2_1112) <- gsub("DR[1-9]", "", names(foods2_1112))
foods1_1112 <- foods1_1112[c(1, 15:51)]
foods2_1112 <- foods2_1112[c(1, 15:51)]

##2011-2012 rbind food
foods1112 <- rbind(foods1_1112, foods2_1112)
foods1112 <- data.frame(apply(foods1112, 2, as.numeric))
foods1112 <- stats::aggregate(. ~ SEQN, data = foods1112, FUN = "mean")
names(foods1112) <- gsub("T_", "", names(foods1112))

##combine food and nutrient data + demographic data
diet1112 <- merge(foods1112, nutrients1112, by = "SEQN")
diet1112 <- merge(demo_1112, diet1112, by = "SEQN")

attach(diet1112)
old_diet1112 <- diet1112[ which(RIDAGEYR >= 60),]
detach(diet1112)

cd1112 <- merge(diet1112, cog1112, by = "SEQN")

##Get diabetes questionnaire
dm1112 <- nhanes("DIQ_G")

#merge with CD DF
cd1112 <- merge(cd1112, dm1112, by = "SEQN")

#Get subjetive memory question
mem1112 <- nhanes('MCQ_G')
mem1112 <- mem1112[c(1,13)]
cd1112 <- merge(cd1112, mem1112, by = 'SEQN')

#cd1112 reference file
write.csv(cd1112, file = "NHANES 11-12 Diet and Cog Data.csv", row.names = F)
cd1112 <- read.csv(file.choose())



### ----------NHANES 2013-2014 data correlates with the letter "H"---------- ###
library(nhanesA)

##load NHANES cog data
cog1314 <- nhanes('CFQ_H')
```

```
##load NHANES 2013-2014 nutrient data
nutrients1_1314 <- nhanes('DR1TOT_H')
nutrients2_1314 <- nhanes('DR2TOT_H')

foods1_1314 <- read_sas(file.choose())
foods2_1314 <- read_sas(file.choose())

coded.food1.1314 <- nhanes('DR1IFF_H')
coded.food2.1314 <- nhanes('DR2IFF_H')
foodcode1314 <- nhanes('DRXFCD_H')

#select only olive oil from individual food data
coded.food1.1314 <- coded.food1.1314[c(1, 18, 19)]
coded.food1.1314 <- coded.food1.1314[ which(coded.food1.1314$DR1IFDCD ==
82104000), ]

coded.food2.1314 <- coded.food2.1314[c(1, 18, 19)]
colnames(coded.food2.1314)[colnames(coded.food2.1314)=="DR2IFDCD"] <-
"DR1IFDCD"
colnames(coded.food2.1314)[colnames(coded.food2.1314)=="DR2IGRMS"] <-
"DR1IGRMS"
coded.food2.1314 <- coded.food2.1314[ which(coded.food2.1314$DR1IFDCD ==
82104000), ]

#combine coded food DFs
coded.food1314 <- rbind(coded.food1.1314, coded.food2.1314)
colnames(coded.food1314)[colnames(coded.food1314)=="DR1IGRMS"] <- "OliveOil.g"
coded.food1314 <- coded.food1314[-c(2)]
coded.food1314 <- stats::aggregate(. ~ SEQN, data = coded.food1314, FUN = "mean")

##2013 - 2014 nutrient data processing
names(nutrients1_1314) <- gsub("DR[1-9]", "", names(nutrients1_1314))
nutrients1_1314 <- nutrients1_1314[, names(nutrients1_1314) %in% nutr_keepers]
names(nutrients2_1314) <- gsub("DR[1-9]", "", names(nutrients2_1314))
nutrients2_1314 <- nutrients2_1314[, names(nutrients2_1314) %in% nutr_keepers]
nutrients1314 <- rbind(nutrients1_1314, nutrients2_1314)
nutrients1314 <- data.frame(apply(nutrients1314, 2, as.numeric))
nutrients1314 <- nutrients1314[!is.na(nutrients1314$TSFAT), ]
nutrients1314 <- stats::aggregate(. ~ SEQN, data = nutrients1314, FUN = "mean")

##load NHANES demographic data
demo_1314 <- nhanes("DEMO_H")

##2013 - 2014 change food var names
names(foods1_1314) <- gsub("DR[1-9]", "", names(foods1_1314))
names(foods2_1314) <- gsub("DR[1-9]", "", names(foods2_1314))
```

```
foods1_1314 <- foods1_1314[c(1, 15:51)]
foods2_1314 <- foods2_1314[c(1, 15:51)]

##2013 - 2014 rbind food
foods1314 <- rbind(foods1_1314, foods2_1314)
foods1314 <- data.frame(apply(foods1314, 2, as.numeric))
foods1314 <- stats::aggregate(. ~ SEQN, data = foods1314, FUN = "mean")
names(foods1314) <- gsub("T_", "", names(foods1314))

##combine food and nutrient data + demographic data
diet1314 <- merge(foods1314, nutrients1314, by = "SEQN")
diet1314 <- merge(demo_1314, diet1314, by = "SEQN")

attach(diet1314)
old_diet1314 <- diet1314[ which(RIDAGEYR >= 60),]
detach(diet1314)

cd1314 <- merge(diet1314, cog1314, by = "SEQN")

##Get diabetes questionnaire
dm1314 <- nhanes("DIQ_H")

#merge with CD DF
cd1314 <- merge(cd1314, dm1314, by = "SEQN")

#Get subjetive memory question
mem1314 <- nhanes('MCQ_H')
mem1314 <- mem1314[c(1,13)]
cd1314 <- merge(cd1314, mem1314, by = 'SEQN')

#cd1314 reference file
write.csv(cd1314, file = "NHANES 13-14 Diet and Cog Data.csv", row.names = F)
cd1314 <- read.csv(file.choose())



### ----------Combine cd1112 & cd1314---------- ###
cd.combined <- rbind(cd1112, cd1314)

# reference file for cd.combined
write.csv(cd.combined, file = "Combined CD Data.csv", row.names = F)

# replace prediabetes 'NA' with '2'
library(tidyr)
library(plyr)
cd.combined$Prediabetes <- replace_na(cd.combined$Prediabetes, 2)
```

```
# recode DM
cd.combined$Diabetes <- factor(cd.combined$Diabetes)
cd.combined$Diabetes <- revalue(cd.combined$Diabetes, c('1' = "yes", '2' = "no"))

# recode PreDM
cd.combined$Prediabetes <- factor(cd.combined$Prediabetes)
cd.combined$Prediabetes <- revalue(cd.combined$Prediabetes, c('1' = "yes", '2' =
"no"))

#add in olive oil variable
coded.food <- rbind(coded.food1112, coded.food1314)
cd.combined <- merge(cd.combined, coded.food, by = 'SEQN', all.x = T)
cd.combined$OliveOil.g <- replace_na(cd.combined$OliveOil.g, 0)

#Investigate caloric intake#
range(cd.combined$TKCAL)
library(spatialEco)
cd.combined$mod.z <- outliers(cd.combined$TKCAL)
cd.combined$mod.z
cd.combined[ which(cd.combined$mod.z > 3.2), ]
cd.combined[ which(cd.combined$mod.z < -1.2), ]
# so far have not removed any outliers


#----------Calculate Mediterranean diet scores----------#
# User-built function based off of Sofi et. al as described in manuscript #
library(KUMC.Med.Diet)
Sofi <- FPEDSofi(cd.combined,
        Fruit = F_CITMLB + F_OTHER,
        Vegetables = V_DRKGR + V_REDOR_TOMATO + V_REDOR_OTHER +
V_STARCHY_OTHER,
        Legumes = PF_LEGUMES,
        Cereals = G_WHOLE,
        Fish = PF_SEAFD_LOW + PF_SEAFD_HI,
        Meat = PF_MEAT + PF_CUREDMEAT + PF_ORGAN,
        Dairy = D_TOTAL,
        Alcohol = A_DRINKS,
        OliveOil = OliveOil.g)

cd.combined$MediterraneanScore <- as.numeric(unlist(Sofi$Absolute))

#Tertiles by Med Adherence
cd.combined <- mutate(cd.combined, Med.Tertile =
ntile(cd.combined$MediterraneanScore,3))
cd.combined$Med.Tertile <- factor(cd.combined$Med.Tertile)
cd.combined$Med.Tertile <- revalue(cd.combined$Med.Tertile, c('1' = 'Low', '2' =
'Medium',
```

```
                                        '3' = 'High'))


# ----------Calculate education-dependent cog z-scores---------- #
education1 <- cd.combined[ which(cd.combined$Education == '<9th Grade'), ]
education2 <- cd.combined[ which(cd.combined$Education == '9-11th Grade'), ]
education3 <- cd.combined[ which(cd.combined$Education == 'High School/GED'), ]
education4 <- cd.combined[ which(cd.combined$Education == 'Some College/AA
Degree'), ]
education5 <- cd.combined[ which(cd.combined$Education == 'College Graduate or
Above'), ]

### Education Classification 1 ###
ed1 <- svydesign(ids = ~SDMVPSU,
        strata = ~SDMVSTRA,
        weights = ~MEC4YR,
        nest = TRUE,
        data = education1)

svymean(~DigitSymbol_Score, ed1, na.rm = T) #28.575
svysd(~DigitSymbol_Score, ed1, na.rm = T) #13.81
education1$digit.z.ed <- ((education1$DigitSymbol_Score - 28.575)/13.81)

svymean(~CERAD_avg, ed1, na.rm = T) #5.2603
svysd(~CERAD_avg, ed1, na.rm = T) #1.595
education1$CERAD.z.ed <- ((education1$CERAD_avg - 5.2603)/1.595)

svymean(~CERAD_Delayed_Recall, ed1, na.rm = T) #4.7727
svysd(~CERAD_Delayed_Recall, ed1, na.rm = T) #2.736
education1$CERAD_delayed.z.ed <- ((education1$CERAD_Delayed_Recall - 4.7727)/
2.736)

svymean(~AnimalFluency_Score, ed1, na.rm = T) #18.05
svysd(~AnimalFluency_Score, ed1, na.rm = T) #5.708
education1$AFT.z.ed <- ((education1$AnimalFluency_Score - 13.931)/4.591)


### Education Classification 2 ###
ed2 <- svydesign(ids = ~SDMVPSU,
        strata = ~SDMVSTRA,
        weights = ~MEC4YR,
        nest = TRUE,
        data = education2)

svymean(~DigitSymbol_Score, ed2, na.rm = T) #40.923
svysd(~DigitSymbol_Score, ed2, na.rm = T) #13.654
education2$digit.z.ed <- ((education2$DigitSymbol_Score - 40.923)/13.654)
```

```
svymean(~CERAD_avg, ed2, na.rm = T) #5.9407
svysd(~CERAD_avg, ed2, na.rm = T) #1.467
education2$CERAD.z.ed <- ((education2$CERAD_avg - 5.9407)/1.467)

svymean(~CERAD_Delayed_Recall, ed2, na.rm = T) #5.4743
svysd(~CERAD_Delayed_Recall, ed2, na.rm = T) #2.134
education2$CERAD_delayed.z.ed <- ((education2$CERAD_Delayed_Recall - 5.4743)/
2.134)

svymean(~AnimalFluency_Score, ed2, na.rm = T) #15.019
svysd(~AnimalFluency_Score, ed2, na.rm = T) #4.503
education2$AFT.z.ed <- ((education2$AnimalFluency_Score - 15.019)/4.503)

### Education Classification 3 ###
ed3 <- svydesign(ids = ~SDMVPSU,
              strata = ~SDMVSTRA,
              weights = ~MEC4YR,
              nest = TRUE,
              data = education3)

svymean(~DigitSymbol_Score, ed3, na.rm = T) #48.787
svysd(~DigitSymbol_Score, ed3, na.rm = T) #14.796
education3$digit.z.ed <- ((education3$DigitSymbol_Score - 48.787)/14.796)

svymean(~CERAD_avg, ed3, na.rm = T) #6.378
svysd(~CERAD_avg, ed3, na.rm = T) #1.486
education3$CERAD.z.ed <- ((education3$CERAD_avg - 6.378)/1.486)

svymean(~CERAD_Delayed_Recall, ed3, na.rm = T) #5.9109
svysd(~CERAD_Delayed_Recall, ed3, na.rm = T) #2.228
education3$CERAD_delayed.z.ed <- ((education3$CERAD_Delayed_Recall - 5.9109)/
2.228)

svymean(~AnimalFluency_Score, ed3, na.rm = T) #16.309
svysd(~AnimalFluency_Score, ed3, na.rm = T) #4.654
education3$AFT.z.ed <- ((education3$AnimalFluency_Score - 16.309)/4.654)

### Education Classification 4 ###
ed4 <- svydesign(ids = ~SDMVPSU,
              strata = ~SDMVSTRA,
              weights = ~MEC4YR,
              nest = TRUE,
              data = education4)

svymean(~DigitSymbol_Score, ed4, na.rm = T) #55.407
svysd(~DigitSymbol_Score, ed4, na.rm = T) #15.091
```

```
education4$digit.z.ed <- ((education4$DigitSymbol_Score - 55.407)/15.091)

svymean(~CERAD_avg, ed4, na.rm = T) #6.712
svysd(~CERAD_avg, ed4, na.rm = T) #1.441
education4$CERAD.z.ed <- ((education4$CERAD_avg - 6.712)/1.441)

svymean(~CERAD_Delayed_Recall, ed4, na.rm = T) #6.5149
svysd(~CERAD_Delayed_Recall, ed4, na.rm = T) #2.285
education4$CERAD_delayed.z.ed <- ((education4$CERAD_Delayed_Recall - 6.5149)/
2.285)

svymean(~AnimalFluency_Score, ed4, na.rm = T) #18.483
svysd(~AnimalFluency_Score, ed4, na.rm = T) #5.22
education4$AFT.z.ed <- ((education4$AnimalFluency_Score - 18.483)/5.22)

### Education Classification 5 ###
ed5 <- svydesign(ids = ~SDMVPSU,
            strata = ~SDMVSTRA,
            weights = ~MEC4YR,
            nest = TRUE,
            data = education5)

svymean(~DigitSymbol_Score, ed5, na.rm = T) #59.824
svysd(~DigitSymbol_Score, ed5, na.rm = T) #14.261
education5$digit.z.ed <- ((education5$DigitSymbol_Score - 59.824)/14.261)

svymean(~CERAD_avg, ed5, na.rm = T) #6.9272
svysd(~CERAD_avg, ed5, na.rm = T) #1.482
education5$CERAD.z.ed <- ((education5$CERAD_avg - 6.9272)/1.482)

svymean(~CERAD_Delayed_Recall, ed5, na.rm = T) #6.6285
svysd(~CERAD_Delayed_Recall, ed5, na.rm = T) #2.28
education5$CERAD_delayed.z.ed <- ((education5$CERAD_Delayed_Recall - 6.6285)/
2.28)

svymean(~AnimalFluency_Score, ed5, na.rm = T) #20.726
svysd(~AnimalFluency_Score, ed5, na.rm = T) #6.054
education5$AFT.z.ed <- ((education5$AnimalFluency_Score - 20.726)/6.054)

# Subset, rbind, and merge education strata #
education1 <- education1[c(1, 183:186)]
education2 <- education2[c(1, 183:186)]
education3 <- education3[c(1, 183:186)]
education4 <- education4[c(1, 183:186)]
education5 <- education5[c(1, 183:186)]

education <- rbind(education1, education2, education3, education4, education5)
```

```
cd.combined <- merge(cd.combined, education, by = 'SEQN', all.x = T)

# Calculate education adjusted global score #
cd.combined$global.z.ed <- ((cd.combined$digit.z.ed + cd.combined$CERAD.z.ed +
                        cd.combined$CERAD_delayed.z.ed + cd.combined$AFT.z.ed)/4)

# Assign high and low cognition status #
cd.combined$digit.status <- ifelse(cd.combined$digit.z.ed <= -1, 1, 0)
cd.combined$cerad.status <- ifelse(cd.combined$CERAD.z.ed < -1, 1, 0)
cd.combined$delayed.status <- ifelse(cd.combined$CERAD_delayed.z.ed < -1, 1, 0)
cd.combined$aft.status <- ifelse(cd.combined$AFT.z.ed < -1, 1, 0)
cd.combined$global.status <- ifelse(cd.combined$global.z.ed < -1, 1, 0)


# ----------recode values---------- #
cd.combined$Cycle <- factor(cd.combined$Cycle)
cd.combined$Gender <- factor(cd.combined$Gender)
cd.combined$Gender <- revalue(cd.combined$Gender, c('1' = "Male", '2' = "Female"))
cd.combined$Race <- factor(cd.combined$Race)
cd.combined$Race <- revalue(cd.combined$Race, c('1' = "Mexican American",
                              '2' = "Other Hispanic",
                              '3' = 'Non-Hispanic White',
                              '4' = 'Non-Hispanic Black',
                              '6' = 'Non-Hispanic Asian',
                              '7' = 'Other Race'))

cd.combined$Birth_Country <- factor(cd.combined$Birth_Country)
cd.combined$Birth_Country <- revalue(cd.combined$Birth_Country, c('1' = 'United
States',
                                         '2' = 'Other',
                                         '77' = 'Refused',
                                         '99' = 'IDK'))

cd.combined$Citizen <- factor(cd.combined$Citizen)
cd.combined$Citizen <- revalue(cd.combined$Citizen, c('1' = 'Citizen',
                                 '2' = 'Not Citizen',
                                 '7' = 'Refused',
                                 '9' = 'IDK'))

cd.combined$Education <- factor(cd.combined$Education)
cd.combined$Education <- revalue(cd.combined$Education, c('1' = '<9th Grade',
                         '2' = '9-11th Grade',
                         '3' = 'High School/GED',
                         '4' = 'Some College/AA Degree',
                         '5' = 'College Graduate or Above',
                         '7' = 'Refused',
                         '9' = 'IDK'))
```

```
cd.combined$Marital_Status <- factor(cd.combined$Marital_Status)
cd.combined$Marital_Status <- revalue(cd.combined$Marital_Status, c('1' = 'Married',
                                                  '2' = 'Widowed',
                                                  '3' = 'Divorced',
                                                  '4' = 'Separated',
                                                  '5' = 'Never Married',
                                                  '6' = 'Live with Partner',
                                                  '77' = 'Refused',
                                                  '99' = 'IDK'))

cd.combined$Annual_Household_Income <-
factor(cd.combined$Annual_Household_Income)
cd.combined$Annual_Household_Income <-
revalue(cd.combined$Annual_Household_Income, c('1' = '$0-4,999',
                                               '2' = '$5,000-9,999',
                                               '3' = '$10,000-14,999',
                                               '4' = '$15,000-19,999',
                                               '5' = '$20,000-24,999',
                                               '6' = '$25,000-34,999',
                                               '7' = '$35,000-44,999',
                                               '8' = '$45,000-54,999',
                                               '9' = '$55,000-64,999',
                                               '10' = '$65,000-74,999',
                                               '12' = '$20,000 and Over',
                                               '13' = '$20,000 and Under',
                                               '14' = '$75,000-99,999',
                                               '15' = '$100,000 and Over',
                                               '77' = 'Refused',
                                               '99' = 'IDK'))



# ----------merge additional covariate data---------- #
#merge cd.combine with BMI
BMI11 <- nhanes("BMX_G")
BMI13 <- nhanes("BMX_H")

BMI.merge <- rbind(BMI11, BMI13)
BMI.merge <- BMI.merge[c(1,11)]
BMI.merge$BMI <- BMI.merge$BMXBMI
BMI.merge <- BMI.merge[c(1,3)]

cd.combined <- merge(cd.combined, BMI.merge, by = "SEQN", all.x = T)

#merge cd.combined with smoking status
smo11 <- nhanes("SMQ_G")
```

```
smo13 <- nhanes("SMQ_H")
smo11 <- smo11[c(1, 2)]
smo13 <- smo13[c(1, 2)]

smo.merge <- rbind(smo11, smo13)
smo.merge$smoke <- smo.merge$SMQ020
smo.merge <- smo.merge[c(1,3)]

cd.combined <- merge(cd.combined, smo.merge, by = "SEQN", all.x = T)
cd.combined$smoke <- factor(cd.combined$smoke)
cd.combined$smoke <- revalue(cd.combined$smoke, c('1' = "Yes", '2' = "No", '7' =
'Refused',
                                        '9' = "IDK"))

#scrape for CVD, stroke, and HTN variables#
mcq1112 <- nhanes('MCQ_G')
mcq1314 <- nhanes('MCQ_H')

cvd.vars <- c('SEQN', 'MCQ160B', 'MCQ160C', 'MCQ160D', 'MCQ160E', 'MCQ160F')

mcq1112 <- mcq1112[cvd.vars]
mcq1314 <- mcq1314[cvd.vars]

mcq <- rbind(mcq1112, mcq1314)

mcq$MCQ160B <- ifelse(mcq$MCQ160B == 'Yes', 1, 0)
mcq$MCQ160B <- mcq$MCQ160B %>% replace_na('No')
colnames(mcq)[2] <- 'hrt.failure'

mcq$MCQ160C <- ifelse(mcq$MCQ160C == 'Yes', 1, 0)
mcq$MCQ160C <- mcq$MCQ160C %>% replace_na('No')

mcq$MCQ160D <- ifelse(mcq$MCQ160D == 'Yes', 1, 0)
mcq$MCQ160D <- mcq$MCQ160D %>% replace_na('No')

mcq$MCQ160E <- ifelse(mcq$MCQ160E == 'Yes', 1, 0)
mcq$MCQ160E <- mcq$MCQ160E %>% replace_na('No')

mcq$MCQ160F <- ifelse(mcq$MCQ160F == 1, 'Yes', 'No')
mcq$MCQ160F <- mcq$MCQ160F %>% replace_na('No')

mcq <- mcq[-c(7)]
mcq <- mcq %>% mutate(CVD = ifelse(Reduce(`|`, lapply(.[,c(2:5)], `==`, 1)), 'Yes', 'No'))
mcq <- mcq[c(1,6,7)]
colnames(mcq)[2] <- 'Stroke'

cd.combined <- merge(cd.combined, mcq, by = 'SEQN')
```

```
htn1112 <- nhanes('BPQ_G')
htn1314 <- nhanes('BPQ_H')

htn.vars <- c('SEQN', 'BPQ020')
htn1112 <- htn1112[htn.vars]
htn1314 <- htn1314[htn.vars]
htn <- rbind(htn1112, htn1314)
colnames(htn)[2] <- 'Hypertension'
htn$Hypertension <- ifelse(htn$Hypertension == 1, 'Yes', 'No')
htn$Hypertension <- htn$Hypertension %>% replace_na('No')
htn$Hypertension <- factor(htn$Hypertension)

cd.combined <- merge(cd.combined, htn, by = 'SEQN')


## ----------Population survey weighting---------- ##
library(survey)
library(jtools)
library(multcomp)
cd.combined$MEC4YR <- .5 * cd.combined$WTMEC2YR

#Build full sample [survey] object using 4-year weightings and NHANES strata
design <- svydesign(ids = ~SDMVPSU,
                strata = ~SDMVSTRA,
                weights = ~MEC4YR,
                nest = TRUE,
                data = cd.combined)



## ----------Entire Sample Analysis---------- ##
svyhist(~MediterraneanScore, design)
svyhist(~CERAD.z, design)
svyhist(~digit.z, design)
svyhist(~AFT.z, design)
svyhist(~CERAD_delayed.z, design)
svyhist(~global.z, design)

#OLS of cog scores and continuous MedD score
summ(svyglm(CERAD.ed.z~MediterraneanScore + Age + Gender + BMI + Race +
Education
            + Marital_Status + FI_Poverty_Ratio + Diabetes + smoke, design=design),
    confint = T, digits = 3)

summ(svyglm(digit..ed.z~MediterraneanScore + Age + Gender + BMI + Race +
Education
```

```
            + Marital_Status + FI_Poverty_Ratio + Diabetes + smoke, design=design),
    confint = T, digits = 3)

summ(svyglm(AFT.ed.z~MediterraneanScore + Age + Gender + BMI + Race +
Education
            + Marital_Status + FI_Poverty_Ratio + Diabetes + smoke, design=design),
    confint = T, digits = 3)

summ(svyglm(CERAD_delayed.ed.z~MediterraneanScore + Age + Gender + BMI +
Race + Education
            + Marital_Status + FI_Poverty_Ratio + Diabetes + smoke, design=design),
    confint = T, digits = 3)

summ(svyglm(global.ed.z~MediterraneanScore + Age + Gender + BMI + Race +
Education
            + Marital_Status + FI_Poverty_Ratio + Diabetes + smoke, design=design),
    confint = T, digits = 3)

#kernel smoothing of global cognition by med score
plot(svysmooth(CERAD.ed.z~MediterraneanScore, design, bandwidth = .5))
plot(svysmooth(digit.ed.z~MediterraneanScore, design, bandwidth = .5))
plot(svysmooth(AFT.ed.z~MediterraneanScore, design, bandwidth = .5))
plot(svysmooth(CERAD_delayed.ed.z~MediterraneanScore, design, bandwidth = .5))
plot(svysmooth(global.ed.z~MediterraneanScore, design, bandwidth = .5))


#OLS of cog scores and categorical MedD score
summ(glht(svyglm(CERAD.ed.z~Med.Tertile + Age + Gender + BMI + Race + Education
                + Marital_Status + FI_Poverty_Ratio + Diabetes + smoke, design=design),
          linfct = mcp(Med.Tertile = "Tukey")))

summ(glht(svyglm(digit.ed.z~Med.Tertile + Age + Gender + BMI + Race + Education
                + Marital_Status + FI_Poverty_Ratio + Diabetes + smoke, design=design),
          linfct = mcp(Med.Tertile = "Tukey")))

summ(glht(svyglm(AFT.ed.z~Med.Tertile + Age + Gender + BMI + Race + Education
                + Marital_Status + FI_Poverty_Ratio + Diabetes + smoke, design=design),
          linfct = mcp(Med.Tertile = "Tukey")))

summ(glht(svyglm(CERAD_delayed.ed.z~Med.Tertile + Age + Gender + BMI + Race +
Education
                + Marital_Status + FI_Poverty_Ratio + Diabetes + smoke, design=design),
          linfct = mcp(Med.Tertile = "Tukey")))

summ(glht(svyglm(global.ed.z~Med.Tertile + Age + Gender + BMI + Race + Education
                + Marital_Status + FI_Poverty_Ratio + Diabetes + smoke, design=design),
          linfct = mcp(Med.Tertile = "Tukey")))
```

```
# Binary logistic regression and categorical MedD score #
z1 <- svyglm(digit.status~Med.Tertile + Age + Gender + BMI + Race +
        Marital_Status + FI_Poverty_Ratio + Diabetes + Hypertension +
        CVD + Stroke + smoke, design=design, family = 'quasibinomial')
summ(z1)
exp(cbind(OR = coef(z1), confint(z1)))


z2 <- svyglm(cerad.status~Med.Tertile + Age + Gender + BMI + Race +
        Marital_Status + FI_Poverty_Ratio + Diabetes + Hypertension +
        CVD + Stroke + smoke, design=design, family = 'quasibinomial')
summ(z2)
exp(cbind(OR = coef(z2), confint(z2)))


z3 <- svyglm(delayed.status~Med.Tertile + Age + Gender + BMI + Race +
        Marital_Status + FI_Poverty_Ratio + Diabetes + Hypertension +
        CVD + Stroke + smoke, design=design, family = 'quasibinomial')
summ(z3)
exp(cbind(OR = coef(z3), confint(z3)))


z4 <- svyglm(aft.status~Med.Tertile + Age + Gender + BMI + Race +
        Marital_Status + FI_Poverty_Ratio + Diabetes + Hypertension +
        CVD + Stroke + smoke, design=design, family = 'quasibinomial')
summ(z4)
exp(cbind(OR = coef(z4), confint(z4)))


z5 <- svyglm(global.status~Med.Tertile + Age + Gender + BMI + Race +
        Marital_Status + FI_Poverty_Ratio + Diabetes + Hypertension +
        CVD + Stroke + smoke, design=design, family = 'quasibinomial')
summ(z5)
exp(cbind(OR = coef(z5), confint(z5)))


#descriptives for table
svymean(~Marital_Status, design)
svyby(~Marital_Status, ~Med.Tertile, design, svymean, na.rm = T)
svymean(~ex.min, design, na.rm = T)
svyby(~ex.min, ~Med.Tertile, design, svymean, na.rm = T)
svymean(~Education, design)
svyby(~Education, ~Med.Tertile, design, svymean, na.rm = T)
svymean(~Race, design)
svyby(~Race, ~Med.Tertile, design, svymean, na.rm = T)
svymean(~Diabetes, design)
```

```
svyby(~Diabetes, ~Med.Tertile, design, svymean, na.rm = T)
svymean(~smoke, design)
svyby(~smoke, ~Med.Tertile, design, svymean, na.rm = T)
svymean(~Age, design)
svyby(~Age, ~Med.Tertile, design, svymean, na.rm = T)
svymean(~Gender, design)
svyby(~Gender, ~Med.Tertile, design, svymean, na.rm = T)
svymean(~BMI, design)
svyby(~BMI, ~Med.Tertile, design, svymean, na.rm = T)
svymean(~FI_Poverty_Ratio, design)
svyby(~FI_Poverty_Ratio, ~Med.Tertile, design, svymean, na.rm = T)
svymean(~Hypertension, design)
svyby(~Hypertension, ~Med.Tertile, design, svymean, na.rm = T)
svymean(~CVD, design)
svyby(~CVD, ~Med.Tertile, design, svymean, na.rm = T)
svymean(~Stroke, design)
svyby(~Stroke, ~Med.Tertile, design, svymean, na.rm = T)
svymean(~MediterraneanScore, design)
svyby(~MediterraneanScore, ~Med.Tertile, design, svymean, na.rm = T)
svymean(~ex.min, design)
svyby(~ex.min, ~Med.Tertile, design, svymean, na.rm = T)
svymean(~CERAD_total, design, na.rm = T)
svyby(~CERAD_total, ~Med.Tertile, design, svymean, na.rm = T)
svymean(~DigitSymbol_Score, design, na.rm = T)
svyby(~DigitSymbol_Score, ~Med.Tertile, design, svymean, na.rm = T)
svymean(~AnimalFluency_Score, design, na.rm = T)
svyby(~AnimalFluency_Score, ~Med.Tertile, design, svymean, na.rm = T)
svymean(~CERAD_Delayed_Recall, design, na.rm = T)
svyby(~CERAD_Delayed_Recall, ~Med.Tertile, design, svymean, na.rm = T)
svyby(~global.z, ~Med.Tertile, design, svymean, na.rm = T)
svyby(~global.z, ~Med.Tertile, design, svymean, na.rm = T)


#GLM for table statistics
summ(svyglm(Age~Med.Tertile, design=design))
svychisq(~Gender+Med.Tertile, design=design)
svychisq(~Race+Med.Tertile, design=design)
svychisq(~Education+Med.Tertile, design=design)
svychisq(~Marital_Status+Med.Tertile, design=design)
svychisq(~Diabetes+Med.Tertile, design=design)
svychisq(~smoke+Med.Tertile, design=design)
svychisq(~Hypertension+Med.Tertile, design=design)
svychisq(~CVD+Med.Tertile, design=design)
svychisq(~Stroke+Med.Tertile, design=design)
summ(svyglm(BMI~Med.Tertile, design=design), digits = 3)
summ(svyglm(ex.min~Med.Tertile, design=design), digits = 3)
summ(svyglm(FI_Poverty_Ratio~Med.Tertile, design=design), digits = 3)
```

```
#exploring other MedD difference among demo characteristics
barplot(svyby(~MediterraneanScore, ~Race, svymean, design = design, na.rm = T),
        legend = F, ylab = "MedD Score", ylim = c(0,7))

summ(svyglm(MediterraneanScore~Gender, design=design), digits = 3)

barplot(svyby(~MediterraneanScore, ~Education, svymean, design = design, na.rm =
T))
barplot(svyby(~MediterraneanScore, ~Race, svymean, design = design, na.rm = T))
barplot(svyby(~MediterraneanScore, ~Diabetes, svymean, design = design, na.rm = T))
barplot(svyby(~MediterraneanScore, ~smoke, svymean, design = design, na.rm = T))

barplot(svyby(~MediterraneanScore, ~age.cat2, svymean, design = design, na.rm = T))
svyby(~MediterraneanScore, ~age.cat2, svymean, design = design, na.rm = T)
summ(svyglm(MediterraneanScore~age.cat, design=design), digits = 3)

barplot(svyby(~MediterraneanScore, ~age.cat2+Race, svymean, design = design,
na.rm = T),
        legend = T, ylim = c(0,8))
svyby(~MediterraneanScore, ~age.cat2+Race, svymean, design = design, na.rm = T)
summ(svyglm(MediterraneanScore~age.cat2, design=design), digits = 3)

svyby(~MediterraneanScore, ~Race, svymean, design = design, na.rm = T)
summ(svyglm(MediterraneanScore~Race, design=design), digits = 3)

barplot(svyby(~MediterraneanScore, ~Education+Race, svymean, design = design,
na.rm = T),
        legend = F)

svyby(~MediterraneanScore, ~age.cat2, svymean, design = design, na.rm = T, vartype
= "ci")
summ(svyglm(MediterraneanScore~age.cat2, design = design), digits = 3)

svyby(~MediterraneanScore, ~Gender, svymean, design = design, na.rm = T, vartype =
"ci")
summ(svyglm(MediterraneanScore~Gender, design = design), digits = 3)

svyby(~MediterraneanScore, ~age.cat2+Race, svymean, design = design, na.rm = T)
summ(svyglm(MediterraneanScore~Race, design = design), digits = 3)

svyby(~MediterraneanScore, ~Education, svymean, design = design, na.rm = T)
summ(svyglm(MediterraneanScore~Education, design = design), digits = 3)

svyby(~MediterraneanScore, ~Marital_Status, svymean, design = design, na.rm = T)
summ(svyglm(MediterraneanScore~Marital_Status, design = design), digits = 3)
```

```
svyby(~MediterraneanScore, ~Diabetes, svymean, design = design, na.rm = T, vartype
= "ci")
summ(svyglm(MediterraneanScore~Diabetes, design = design), digits = 3)

confint(svymean(~DigitSymbol_Score, design, na.rm = T))
confint(svymean(~AnimalFluency_Score, design, na.rm = T))
confint(svymean(~CERAD_total, design, na.rm = T))
confint(svymean(~CERAD_Delayed_Recall, design, na.rm = T))
confint(svymean(~global.z, design, na.rm = T))
svymean(~global.z, design, na.rm = T)

svymean(~Fruit, design, na.rm = T)
svymean(~Vegetables, design, na.rm = T)
svymean(~Legumes, design, na.rm = T)
svymean(~Cereals, design, na.rm = T)
svymean(~Fish, design, na.rm = T)
svymean(~Meat, design, na.rm = T)
svymean(~Dairy, design, na.rm = T)
svymean(~Alcohol, design, na.rm = T)
svymean(~OliveOil, design, na.rm = T)

library(jtools)
svysd(~Fruit, design, na.rm = T)
svysd(~Vegetables, design, na.rm = T)
svysd(~Legumes, design, na.rm = T)
svysd(~Cereals, design, na.rm = T)
svysd(~Fish, design, na.rm = T)
svysd(~Meat, design, na.rm = T)
svysd(~Dairy, design, na.rm = T)
svysd(~Alcohol, design, na.rm = T)
svysd(~OliveOil, design, na.rm = T)

confint(svymean(~Fruit, design, na.rm = T))
confint(svymean(~Vegetables, design, na.rm = T))
confint(svymean(~Legumes, design, na.rm = T))
confint(svymean(~Cereals, design, na.rm = T))
confint(svymean(~Fish, design, na.rm = T))
confint(svymean(~Meat, design, na.rm = T))
confint(svymean(~Dairy, design, na.rm = T))
confint(svymean(~Alcohol, design, na.rm = T))
confint(svymean(~OliveOil, design, na.rm = T))
svyby(~Fruit, ~Med.Tertile, svymean, design = design, na.rm = T)
svyby(~Vegetables, ~Med.Tertile, svymean, design = design, na.rm = T)
svyby(~Legumes, ~Med.Tertile, svymean, design = design, na.rm = T)
svyby(~Cereals, ~Med.Tertile, svymean, design = design, na.rm = T)
svyby(~Fish, ~Med.Tertile, svymean, design = design, na.rm = T)
svyby(~Meat, ~Med.Tertile, svymean, design = design, na.rm = T)
```

```
svyby(~Dairy, ~Med.Tertile, svymean, design = design, na.rm = T)
svyby(~Alcohol, ~Med.Tertile, svymean, design = design, na.rm = T)
svyby(~OliveOil, ~Med.Tertile, svymean, design = design, na.rm = T)
summ(svyglm(Fruit~Med.Tertile, design = design), digits = 3)
summ(svyglm(Vegetables~Med.Tertile, design = design), digits = 3)
summ(svyglm(Legumes~Med.Tertile, design = design), digits = 3)
summ(svyglm(Cereals~Med.Tertile, design = design), digits = 3)
summ(svyglm(Fish~Med.Tertile, design = design), digits = 3)
summ(svyglm(Meat~Med.Tertile, design = design), digits = 3)
summ(svyglm(Dairy~Med.Tertile, design = design), digits = 3)
summ(svyglm(Alcohol~Med.Tertile, design = design), digits = 3)
summ(svyglm(OliveOil~Med.Tertile, design = design), digits = 3)



## ----------Sensitivity Analyses---------- ##
#Create DF with only subjects with no selective memory complaints
cognorm.cd.combined <- cd.combined[ which(cd.combined$MCQ084=='No', )]

#Recalculate MedD adherence tertiles with only sample with no subjective memory
complaints
cognorm.cd.combined <- mutate(cognorm.cd.combined, Med.Tertile =
ntile(cognorm.cd.combined$MediterraneanScore,3))
cognorm.cd.combined$Med.Tertile <- factor(cognorm.cd.combined$Med.Tertile)
cognorm.cd.combined$Med.Tertile <- revalue(cognorm.cd.combined$Med.Tertile, c('1' =
'Low', '2' = 'Medium',

                                                       '3' = 'High'))


#Build sensitivity sample [survey] object using 4-year weightings and NHANES strata
cognorm <- svydesign(ids = ~SDMVPSU,
                strata = ~SDMVSTRA,
                weights = ~MEC4YR,
                nest = TRUE,
                data = cognorm.cd.combined)


# ----------Conduct sensitivity analyses---------- #
n1 <- svyglm(digit.status~Med.Tertile + Age + Gender + BMI + Race +
           Marital_Status + FI_Poverty_Ratio + Diabetes + Hypertension +
           CVD + Stroke + smoke, design=cognorm, family = 'quasibinomial')
summ(n1)
exp(cbind(OR = coef(n1), confint(n1)))


n2 <- svyglm(cerad.status~Med.Tertile + Age + Gender + BMI + Race +
```

```
            Marital_Status + FI_Poverty_Ratio + Diabetes + Hypertension +
            CVD + Stroke + smoke, design=cognorm, family = 'quasibinomial')
summ(n2)
exp(cbind(OR = coef(n2), confint(n2)))



n3 <- svyglm(delayed.status~Med.Tertile + Age + Gender + BMI + Race +
            Marital_Status + FI_Poverty_Ratio + Diabetes + Hypertension +
            CVD + Stroke + smoke, design=cognorm, family = 'quasibinomial')
summ(n3)
exp(cbind(OR = coef(n3), confint(n3)))


n4 <- svyglm(aft.status~Med.Tertile + Age + Gender + BMI + Race +
            Marital_Status + FI_Poverty_Ratio + Diabetes + Hypertension +
            CVD + Stroke + smoke, design=cognorm, family = 'quasibinomial')
summ(n4)
exp(cbind(OR = coef(n4), confint(n4)))


n5 <- svyglm(global.status~Med.Tertile + Age + Gender + BMI + Race +
            Marital_Status + FI_Poverty_Ratio + Diabetes + Hypertension +
            CVD + Stroke + smoke, design=cognorm, family = 'quasibinomial')
summ(n5)
exp(cbind(OR = coef(n5), confint(n5)))


svyby(~global.z, ~Med.Tertile, svymean, design = cognorm, na.rm = T)


svymean(~CERAD.z, cognorm, na.rm = T)
svymean(~digit.z, cognorm, na.rm = T)
svymean(~AFT.z, cognorm, na.rm = T)
svymean(~CERAD_delayed.z, cognorm, na.rm = T)
svymean(~global.z, cognorm, na.rm = T)

svysd(~DigitSymbol_Score, cognorm, na.rm = T)
svysd(~AnimalFluency_Score, cognorm, na.rm = T)
svysd(~CERAD_avg, cognorm, na.rm = T)
svysd(~CERAD_Delayed_Recall, cognorm, na.rm = T)
svysd(~global.z, cognorm, na.rm = T)
svysd(~global.z, cognorm, na.rm = T)

#statistics comparing lowest tertile to highest tertile
summ(svyglm(Fruit~Med.Tertile, design=design),
    confint = T, digits = 3)
```

```
summ(svyglm(Vegetables~Med.Tertile, design=design),
    confint = T, digits = 3)
summ(svyglm(Legumes~Med.Tertile, design=design),
    confint = T, digits = 3)
summ(svyglm(Cereals~Med.Tertile, design=design),
    confint = T, digits = 3)
summ(svyglm(Fish~Med.Tertile, design=design),
    confint = T, digits = 3)
summ(svyglm(Meat~Med.Tertile, design=design),
    confint = T, digits = 3)
summ(svyglm(Dairy~Med.Tertile, design=design),
    confint = T, digits = 3)
summ(svyglm(Alcohol~Med.Tertile, design=design),
    confint = T, digits = 3)
summ(svyglm(OliveOil~Med.Tertile, design=design),
    confint = T, digits = 3)



## ----------[survey] does not calculate survey weigthed SD for subgroups - write code to
calculate---------- ##

# Use tidyverse to build a loop that calculates survey weighted standard deviations by
subgroup #
#This loop is recyclable - this is an example used to calculate SD for MedD components
by MedD adherence tertile
library(tidyverse)
datalist <- list()
for(i in levels(as.factor(cd.combined$Med.Tertile))) {
  df_subset <- cd.combined[cd.combined$Med.Tertile == i, ]
  des <- svydesign(id = ~1 , data = df_subset, weight = ~MEC4YR)
  dat <- data.frame(as.data.frame(sqrt(svyvar(~Fruit, na.rm= TRUE, design = des)))
[,"variance"],
                  as.data.frame(sqrt(svyvar(~Vegetables, na.rm= TRUE, design = des)))
[,"variance"],
                  as.data.frame(sqrt(svyvar(~Legumes, na.rm= TRUE, design = des)))
[,"variance"],
                  as.data.frame(sqrt(svyvar(~Cereals, na.rm= TRUE, design = des)))
[,"variance"],
                  as.data.frame(sqrt(svyvar(~Fish, na.rm= TRUE, design = des)))
[,"variance"],
                  as.data.frame(sqrt(svyvar(~Meat, na.rm= TRUE, design = des)))
[,"variance"],
                  as.data.frame(sqrt(svyvar(~Dairy, na.rm= TRUE, design = des)))
[,"variance"],
                  as.data.frame(sqrt(svyvar(~Alcohol, na.rm= TRUE, design = des)))
[,"variance"],
```

```
                   as.data.frame(sqrt(svyvar(~OliveOil, na.rm= TRUE, design = des)))
[,"variance"])
  colnames(dat)<-c("sd.Fruit", "sd.Veg", "sd.Leg", "sd.Cereals", "sd.Fish", "sd.Meat",
"sd.Dairy",
                  "sd.Alc", "sd.OO")
  datalist[[i]] <- dat
}

df_sd <- as.data.frame(do.call(rbind, datalist)) %>%
  rownames_to_column() %>%
  rename(Med.Tertile = rowname)
df_sd <- t(df_sd)
```
